# Intravascular leiomyomatosis in postmenopausal woman: a case report

**DOI:** 10.3389/fmed.2025.1517261

**Published:** 2025-03-06

**Authors:** Tingting Jiang, Yalan Yang, Li Wang

**Affiliations:** Department of Obstetrics and Gynecology of Changde City First People’s Hospital, Changde, China

**Keywords:** intravenous leiomyomatosis, preoperative diagnosis, etiology, clinical treatment, prognosis

## Abstract

Intravenous leiomyomatosis (IVL) is a rare benign tumor originating from the smooth muscle of the uterus. Tumor cells spread along the veins, which may involve the uterine veins, pelvic veins, inferior vena cava, and even the right atrium. This case report describes a postmenopausal woman with IVL extending into the right heart chambers. The patient successfully underwent surgery and was discharged without complications. This report highlights valuable insights into the preoperative diagnosis and clinical management of IVL.

## Introduction

Intravenous leiomyomatosis (IVL) is a rare mesenchymal tumor that, while histologically benign, exhibits biologically aggressive behavior resembling malignancy (1). Hirschfeld first reported it in 1896, and there have been less than 700 cases documented worldwide (2). IVL typically originates from uterine leiomyomas and can extend through the venous system into the right heart chambers, occasionally leading to life-threatening complications such as sudden cardiac death (3).

Although the exact pathogenesis of IVL remains unclear, two primary hypotheses have been proposed. One hypothesis suggests that IVL arises from smooth muscle cells in the walls of blood vessels, while another hypothesis posits that IVL originates from uterine leiomyoma cells that invade the vessel lumen (4). Recent studies comparing the molecular cytogenetics of IVL and uterine leiomyomas suggest shared chromosomal pathways (5). Early detection and aggressive treatment are essential to prevent severe or fatal outcomes, given the atypical clinical presentation and unpredictable biological behavior of IVL.

## Case presentation

A 55-year-old postmenopausal Chinese woman presented with persistent vaginal bleeding. An initial transvaginal ultrasound at a local hospital suggested pelvic masses presumed to be hydrosalpinx. An endometrial biopsy following curettage revealed proliferative endometrium. In July 2024, the patient was admitted to the Department of Gynecology for further evaluation due to a persistent pelvic mass.

## Physical examination

A bimanual vaginal examination at Changde First People’s Hospital revealed an enlarged uterus and thickened adnexal regions. A cardiovascular examination revealed no significant clinical symptoms, such as fainting, dyspnea, orthopnea, or palpitations. Cardiac auscultation showed no audible murmurs or other abnormalities.

## Imaging and diagnostic findings

**Figure 1:**
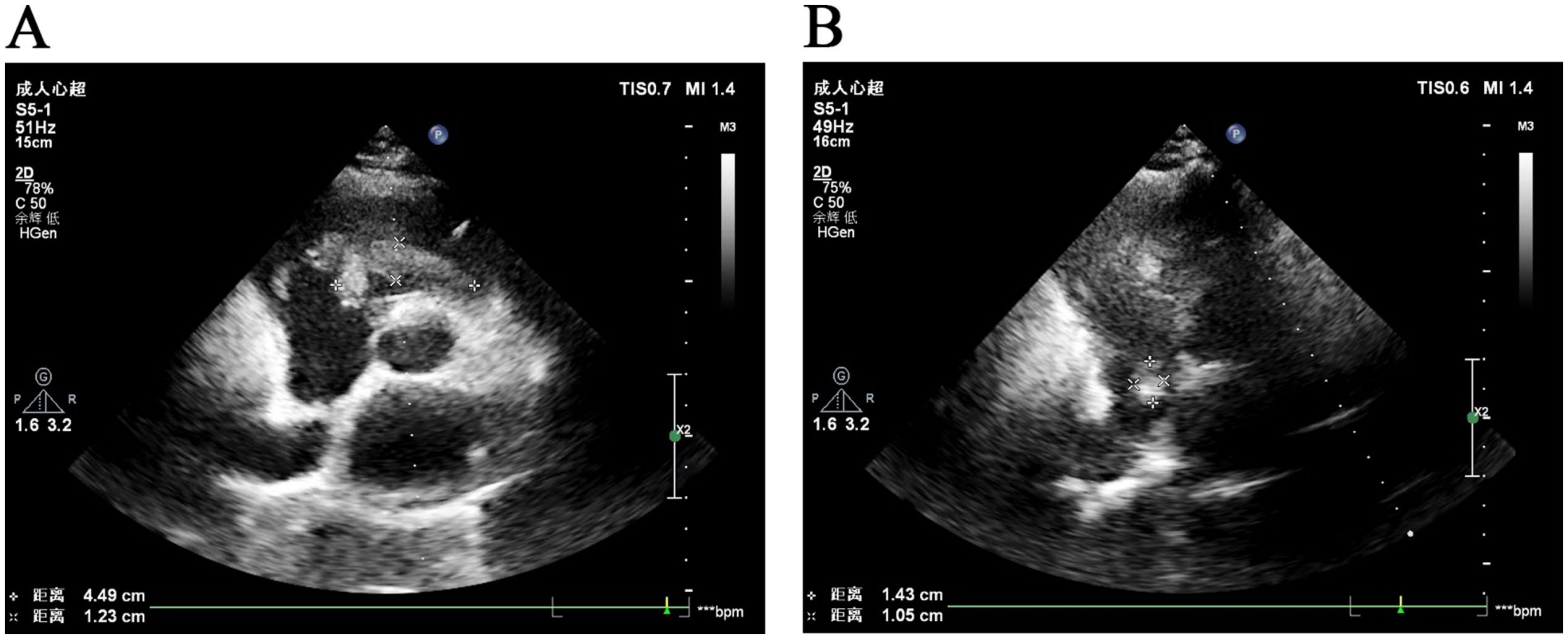
Ultrasonographic images of intravascular leiomyomatosis. **(A)** Ultrasonographic image shows a mixed-echo mass located on the tricuspid valve. **(B)** Ultrasonographic image shows a fibrotic mass near the right atrial septum.

Echocardiography identified a 23 × 13-mm mixed-echo mass near the atrial septum and a 48 × 18-mm mass at the tricuspid valve, with no significant blood flow signals (Figure 1). Transvaginal color Doppler imaging revealed a mixed-echo mass measuring approximately 57 × 28 mm in the right adnexal region with clear boundaries and an irregular shape, which can elicit more abundant dot-stripe blood flow signals. Pelvic MRI confirmed tubular mixed-signal lesions in the right adnexa, with areas of high T1 signal intensity and enhanced margins.

## Surgical treatment

After a multidisciplinary discussion, the patient was finally diagnosed with IVL and underwent combined thoracoabdominal surgery on 22 July 2024. The operation process was jointly presided over by the Department of Cardiovascular Surgery and the Department of Gynecology. Stage I surgery was performed by the Department of Cardiovascular Surgery under cardiopulmonary bypass. The procedure began with a median sternotomy, followed by pericardial incision and suspension. Cardiopulmonary bypass was initiated with vena cava occlusion, systemic cooling, and ascending aortic clamping. Cold perfusion through the aortic root and topical hypothermia using iced saline slush ensured adequate myocardial protection. After satisfactory cardiac arrest, a right atriotomy was performed, revealing fibrous tumors in the tricuspid valve and within the right atrium.

**Figure 2:**
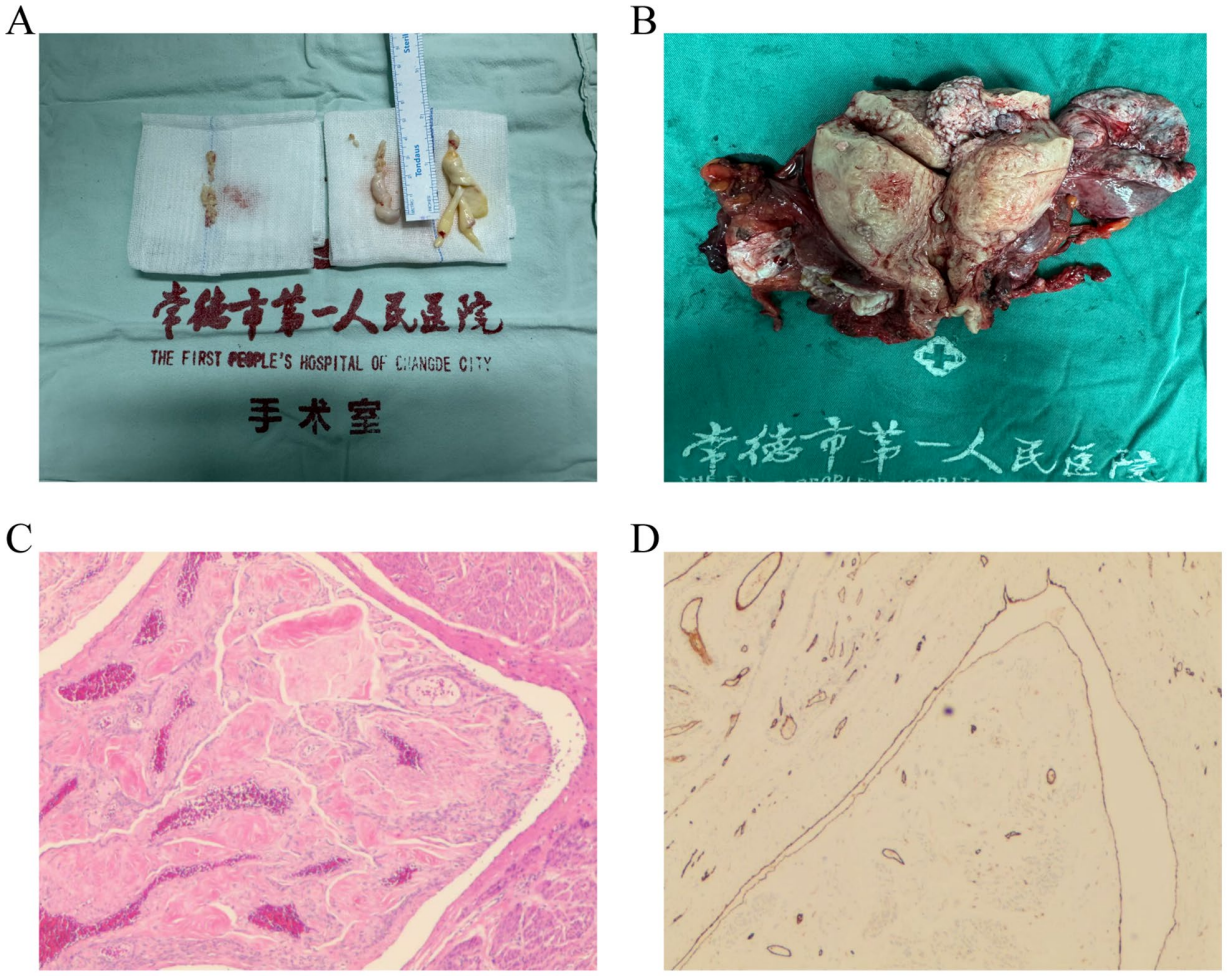
Examples of IVL pathology sections. **(A)** IVL in the right atrium and right ventricle. **(B)** IVL in the uterine vessel. **(C)** HE staining shows irregular blood vessels and leiomyoma tissue. **(D)** Representative immunohistochemical staining shows CD31-positive endothelial cells.

The right atrial tumor measured 1 × 1 × 0.5 cm, and the elongated tumor on the tricuspid valve measured 0.5 × 0.8 × 7 cm (Figure 2A), extending into the right ventricular outflow tract. Both tumors were smooth and successfully excised. No abnormalities were observed in the right ventricle or outflow tract upon inspection. Saline injection testing confirmed proper closure of the tricuspid valve. After rewarming, left ventricular venting, and successful cardiac resuscitation, the heart resumed beating spontaneously with a regular rhythm and no conduction block. The right atrial incision was closed, cardiopulmonary bypass was weaned, and all cannulas were removed. Protamine sulfate was used to neutralize heparin. Hemostasis was achieved, and the chest was closed in layers with mediastinal and pericardial drainage tubes placed.

Immediately following this, the patient underwent second-stage surgery, which included an open hysterectomy and bilateral salpingo-oophorectomy via laparotomy. This procedure was carried out by the gynecologist in collaboration with the cardiovascular surgeon. Ultimately, the two departments worked together seamlessly to ensure a successful surgical outcome. During Stage II surgery, the uterus was found to be enlarged to a size comparable to a 3+ month pregnancy. The anterior and posterior walls, as well as both parauterine regions, appeared irregular with multiple nodules of varying sizes, ranging from approximately 1 to 5 cm in diameter. A prominent nodule, measuring 5 cm in diameter, was observed protruding from the left uterine horn. In addition, multiple purplish-blue nodules of varying sizes were found in both parauterine regions, extending along the parauterine blood vessels, which were markedly distended and engorged. The bilateral fallopian tubes and ovaries appeared grossly normal. Intraoperatively, the findings were consistent with IVL (Figure 2B).

The patient experienced an uneventful recovery and was discharged 2 weeks post-surgery. Pathological findings, including irregular blood vessels and leiomyoma tissue identified via HE staining (Figure 2C) and CD31-positive immunohistochemical staining highlighting vascular endothelial cells (Figure 2D), revealed the presence of smooth muscle tissue in the right ventricular tumor, the right atrial tumor, and the parauterine vessels. These findings, combined with the clinical and medical history, confirmed the diagnosis of IVL.

## Follow-up and outcome

The patient recovered well after surgery and was prescribed oral itraconazole as postoperative therapy. At 1-month follow-up, no signs of recurrence were observed.

## Discussion

Clinical manifestations and imaging findings of intravascular leiomyomatosis (IVL) are often non-specific in the early stages, leading to a high rate of missed or delayed diagnoses. IVL is a rare benign smooth muscle tumor originating in the uterus that can extend along the venous system to the inferior vena cava and the right side of the heart, which may result in fatal outcomes (6). Although IVL is classified as a benign disease, it exhibits malignant-like behavior and is most commonly observed in women of reproductive age with a history of uterine leiomyomas.

The majority of IVL patients have a history of uterine leiomyoma, myomectomy, or hysterectomy. Several studies have explored the molecular mechanisms underlying the development of both intravascular leiomyomatosis and uterine leiomyomas (5, 7). Molecular cytogenetic analyses have revealed that IVL shares chromosomal pathways with uterine leiomyomas (8). In addition, IVL may be associated with adenomyosis or coexisting adenomyosis and leiomyomas (9).

Clinically, the majority of IVL patients are asymptomatic, although some may present with either a pelvic or cardiac mass. In addition, a significant proportion of patients exhibit coexisting lesions in both regions. The most common symptoms reported include pelvic pain and irregular uterine bleeding (10). Preoperative detection of IVL is particularly challenging due to its rarity and the non-specific nature of patient complaints (11). The majority of patients do not experience significant symptoms until advanced stages, often marked by cardiovascular events. Furthermore, cardiac ultrasonography is not always sensitive enough to detect tumors that do not directly involve the heart. Consequently, imaging has become the primary diagnostic modality for IVL. Enhanced CT and MRI are highly effective in identifying and localizing lesions in the pelvis, abdomen, venous system, and heart (12). Common imaging findings include tumor growth extending into the right atrium or inferior vena cava, as well as filling defects in the ovarian or renal veins. IVL progression can be classified into four stages based on the extent of intravascular tumor invasion. Pathological diagnosis remains the gold standard for confirming IVL. Typical pathological features include mature spindle-shaped smooth muscle cells and blood vessels with mirror-image patterns. Immunohistochemical staining often shows positivity for SMA, ER, PR, and caldesmon, which supports the diagnosis.

Surgical resection is the cornerstone of IVL management, aiming to remove the tumor and prevent recurrence (13). In our case, the patient presented with vaginal bleeding and underwent a combined surgical approach involving cardiac tumor excision and hysterectomy with bilateral salpingo-oophorectomy. This decision was made after carefully weighing the risks and benefits. For patients with IVL confined to the pelvic cavity, the surgical procedure is relatively straightforward and can be performed solely by a gynecologist. However, in Stage II or more advanced cases, the surgery becomes more complex and invasive, requiring multidisciplinary collaboration among gynecology, cardiovascular surgery, anesthesiology, and other specialties. Depending on the patient’s condition, a combination of thoracic and abdominal surgery or staged surgical procedures may be necessary (14).

Recent evidence highlights important considerations in the surgical management of leiomyomas, particularly regarding safety, fertility preservation, and long-term outcomes. A meta-analysis comparing laparoscopic and abdominal myomectomy demonstrated that laparoscopic approaches are associated with reduced blood loss, shorter hospital stays, and decreased postoperative pain without increasing complication rates or negatively impacting pregnancy outcomes (15). This finding underscores the importance of individualized surgical planning, especially in patients requiring fertility preservation or managing complex cases such as IVL, where advanced surgical techniques and multidisciplinary collaboration may optimize outcomes. It is also important to consider the risk of misdiagnosing uterine sarcomas during the management of leiomyomas, as sarcomas may initially present with similar clinical and imaging features. Misdiagnosis can lead to suboptimal treatment strategies, such as the use of morcellation, which is associated with a higher risk of recurrence and metastasis in undiagnosed sarcomas. Careful preoperative assessment and the adoption of molecular/genomic profiling could improve tailored management strategies and help identify patients at risk of recurrence (16).

IVL is a hormone-dependent tumor and is generally associated with a favorable prognosis. Tumor progression tends to be more rapid and aggressive in premenopausal women compared to postmenopausal women. For patients unwilling or unable to undergo surgery, or those with large tumors, oral letrozole and similar medications can effectively delay tumor growth, significantly reduce tumor size, and inhibit the recurrence of residual tumors. This pharmacological approach is recommended as an adjunct or alternative to surgery (17).

In brief, our case report emphasizes the clinical features, diagnostic process, and treatment of IVL, providing valuable insights into early diagnosis and management of this rare condition.

## Data Availability

The raw data supporting the conclusions of this article will be made available by the authors, without undue reservation.
